# Vinculin association with actin cytoskeleton is necessary for stiffness-dependent regulation of vinculin behavior

**DOI:** 10.1371/journal.pone.0175324

**Published:** 2017-04-07

**Authors:** Tomohiro Omachi, Takafumi Ichikawa, Yasuhisa Kimura, Kazumitsu Ueda, Noriyuki Kioka

**Affiliations:** 1 Division of Applied Life Sciences, Graduate School of Agriculture, Kyoto University, Sakyo, Kyoto, Japan; 2 Institute for Integrated Cell-Material Sciences (iCeMS), Kyoto University, Sakyo, Kyoto, Japan; The University of Akron, UNITED STATES

## Abstract

The extracellular matrix (ECM) is a major regulator of cell behavior. Recent studies have indicated the importance of the physical properties of the ECM, including its stiffness, for cell migration and differentiation. Using actomyosin-generated forces, cells pull the ECM and sense stiffness via cell-ECM adhesion structures called focal adhesions (FAs). Vinculin, an actin-binding FA protein, has emerged as a major player in FA-mediated mechanotransduction. Although vinculin is important for sensing ECM stiffness, the role of vinculin binding to actin in the ECM stiffness-mediated regulation of vinculin behavior remains unknown. Here, we show that an actin binding-deficient mutation disrupts the ECM stiffness-dependent regulation of CSB (cytoskeleton stabilization buffer) resistance and the stable localization of vinculin. These results suggest that the vinculin-actin interaction participates in FA-mediated mechanotransduction.

## Introduction

Extracellular microenvironments, such as components of the extracellular matrix (ECM), are major regulators of cell behavior. In addition to the chemical properties of the microenvironment, recent evidence has indicated the importance of its physical properties: ECM stiffness directs the differentiation of mesenchymal stem cells to adipocytes or osteoblasts[1]. Cell migration and tumor progression are also regulated through ECM stiffness [2–4]. Using actomyosin-generated forces, cells pull the ECM via cell-ECM adhesion structures known as focal adhesions (FAs) and subsequently counterbalance the tension to sense ECM stiffness: higher intracellular tension is observed on a rigid ECM than on a soft ECM. At FAs, ECM receptor integrin binds to the ECM, linking it to the actin cytoskeleton through a number of cytoplasmic FA proteins.

Vinculin is a major cytoplasmic FA protein. The loss of vinculin expression has been associated with cardiomyopathy [5] and resistance to anoikis [6]. Vinculin-knockout fibroblasts show less spreading but exhibit enhanced 2D cell migration on the ECM [7]. In contrast, vinculin facilitates 3D cell migration [8]. These observations suggest a pivotal role in the FA-mediated regulation of cell behavior. Vinculin comprises an N-terminal head (Vh) and a C-terminal tail (Vt) domains connected via a proline-rich linker region. Vt binds to actin, paxillin and phosphatidylinositol 4,5-bisphosphate [9] and contributes to the contractile stress generation[10]. Vh associates with talin, another cytoplasmic protein that directly binds to integrin. The linker region interacts with vinexin-family proteins and Arp2/3 [11–13]. Vh also intramolecularly associates with Vt to reduce the affinity of vinculin to talin or actin in its closed (inactive) conformation [14]. Disruption of the Vh-Vt interaction induces vinculin activation (activated (open) vinculin) and increases its affinity to actin and talin [14]. Conversely, associations with actin and talin induce the dissociation of the Vh-Vt interaction, leading to vinculin activation *in vitro* [15, 16]. In cells, vinculin is inactive (closed) in the cytosol, and some vinculin is active at FAs [17].

Vinculin binding to actin contributes to the major functions of vinculin, such as the regulation of traction forces and cell migration [18]. Models for the Vt-actin structure have been proposed using electron microscopy [18, 19], but this structure remains controversial due to the limited resolution. A recent study using cryo-electron microscopy indicated the displacement of one of five helices in the Vt helical bundle to mediate interactions with actin [20]. In all models, Vt binds to two actin protomers in F-actin via two surfaces.

Increasing evidence has suggested an association between vinculin and FA-mediated mechanotransduction. Vinculin recruitment to FAs depends on ECM stiffness and intracellular tension [21]. Vinculin binding to actin slows the actin retrograde flow and contributes to the generation of high ECM traction forces [22]. Variations in the amount of tension have been observed within the vinculin molecule at FAs [23]. In addition, vinculin is necessary for ECM stiffness-directed myoblast or adipocyte differentiation [24, 25]. In a previous study, we also showed that a stiff ECM promotes interactions between the vinculin linker region and vinexin α, resulting in the vinculin localization to lipid rafts [26] and the vinculin activation [27], thus stable localization of vinculin at FAs and vinculin resistance to CSB (cytoskeleton stabilization buffer). Furthermore, the loss of vinexin expression impairs ECM stiffness-dependent cell migration [27], indicating that the interaction of vinculin with vinexin α functions as a sensor for ECM stiffness. Although several reports have suggested a role for the vinculin interaction with actin in vinculin activation, it is not clear whether the interaction with actin is involved in the ECM stiffness-dependent regulation of vinculin behavior. Here, we examined a role for vinculin interaction with actin in the ECM stiffness-dependent regulation of vinculin behavior using actin binding-deficient vinculin mutants.

## Materials and methods

### Antibodies and reagents

Mouse anti-vinculin (hVin) and anti-talin (8d4) antibodies were purchased from Sigma Aldrich (St Louis, MO). Mouse anti-paxillin antibody was from Life Technologies (Carlsbad, CA). Type I collagen was purchased from Nitta Gelatin (Osaka, Japan). Puromycin and Blasticidin were obtained from Sigma Aldrich and Kaken Pharmaceutical (Tokyo, Japan), respectively.

### Plasmid and protein purification

Monomeric GFP-tagged vinculin in pCDH-EF1-IRES-blasti vector (System Biosciences, Mountain View, CA) was reported previously [27]. The vinculin IA mutant (I997A) and VA mutant (V1001A), and T12 (D974A:K975A:R976A:R978A) were generated by using In-Fusion Kit (Takara Bio, Ohtsu, Japan). Mutants having both T12 and IA mutations (T12/IA) and T12 and VA mutations (T12/VA) were also generated. pLKO.1 harboring vinculin small hairpin RNA (5’-CCCTGTACTTTCAGTTACTAT-3’) was purchased from Openbiosystems (Waltham, MA). Vinculin and mutants were subcloned into pColdI vector (Takara Bio) for purification. The His-tagged vinculin or GST-VBS(IpaA 610–633 a.a) were expressed in *E*.*coli* Rosetta (MERCK, Darmstadt, Germany) and purified as described previously [27, 28].

### Cell culture and lentivirus infection

Mouse embryonic fibroblasts (MEFs) established in the previous reports [27, 29] were grown in Dulbecco’s Modified Eagle’s Medium (DMEM; Nacalai tesque, Kyoto, Japan) supplemented with 10% fetal bovine serum (FBS; Gibco/Thermo Fisher Scientific, Waltham, MA). Lentivirus were generated using vectors pCDH or pLKO.1 as previously described [27]. Lentivirus harboring vinculin shRNA was infected to wild-type (WT) MEFs first to establish vinculin-knockdown (KD) cells [27]. Vinculin KD cells were further infected with lentiviruses to generate GFP-vinculin-expressing cells. A polyacrylamide gel substrate was prepared as previously described [27, 30]. Gels of 3.8, 7.4, and 25 kPa were used.

### Actin co-sedimentation assay

His-tagged WT vinculin or mutants were incubated with 4 μM pre-polymerized F-actin in the buffer (50 mM Tris-HCl (pH 8.0), 0.2 mM MgCl_2_, 0.2 mM ATP, 1 mM DTT, and protease inhibitor cocktail (Roche, Basel, Switzerland)) containing 25 μM VBS3(talin) peptide or 10 μM GST-VBS(IpaA) for 1 hour at 24°C, followed by ultracentrifugation at 150,000 x g for 1 hour at 4°C. The pellet and half of the supernatant were subjected to SDS-PAGE, followed by CBB staining.

### Quantification of cell area, FAs and CSB-resistant FAs

Immunostaining was performed to visualize and quantify the cell area and the total or CSB-resistant GFP-vinculin as previously described [27]. Briefly, for total vinculin, the cells were fixed with 1.5% paraformaldehyde for 45 min without pretreatment. For CSB-resistant vinculin, the cells were initially treated with CSB (0.5% Triton X-100, 10 mM PIPES, pH 6.8, 50 mM NaCl, 3 mM MgCl_2_, 300 mM sucrose and complete protease inhibitor cocktail (Roche, Basel, Switzerland)) for 1 min at 4°C, followed by fixation with 4% paraformaldehyde for 15 min. The cells were then subjected to immunostaining. Photographs were obtained using an LSM 700 confocal microscopy system equipped with a Plan-Apochromat 40×/1.3 NA oil immersion objective (Carl Zeiss, Oberkochen, Germany). The number of FAs containing vinculin and total integrated density of vinculin in each cell were quantified using ImageJ software. Conditions (gain and laser power) for taking images on gel substrates and cover slips were different, thus it is difficult to compare the results on gels with those on cover slips directly.

### Fluorescence recovery after photobleaching (FRAP) analysis

FRAP studies were performed as previously described [27], with slight modification. Briefly, FRAP measurements were performed at 37°C in FluoroBrite™ DMEM (Thermo Fisher Scientific) supplemented with 10% FBS. Stable FAs near the cell edge were selected as the region of interest (ROI), and four FAs in each cell were photobleached. The fluorescence recovery of the FAs within the ROI was observed every 5 s (50 frames) and subsequently analyzed using ZEN 2009 (Carl Zeiss). A total of eighty FAs were examined across three independent experiments. Normalized FRAP curves were fitted to a single exponential function using Origin 8.5.1 software, and the immobile fraction was calculated as described [27].

### Statistical analysis

Statistical analysis was performed by using one-way ANOVA and the post hoc Scheffe’s test or Bonferroni’s test in Origin 8.5.1software.

## Results

### Actin binding of vinculin mutants

**Fig 1:**
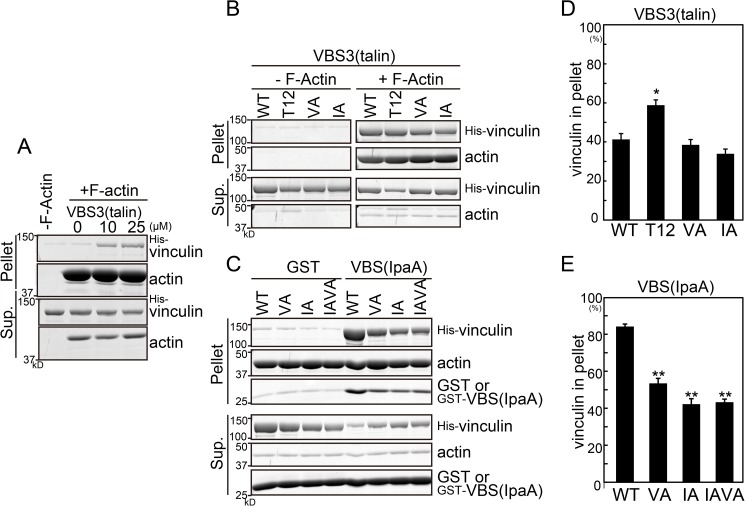
Actin co-sedimentation assay using vinculin mutants. (A) His-tagged WT vinculin (0.25 μM) with VBS3(talin) peptide (0, 10, or 25 μM) was incubated with F-actin, then ultracentrifuged. (B) His-tagged vinculin mutants (2 μM) and 25 μM VBS3(talin) were incubated with or without F-actin, then ultracentrifuged. (C) His-tagged vinculin mutants (1 μM) and 10 μM GST-VBS(IpaA) or GST were incubated with F-actin, then ultracentrifuged (right panel). Loading control (left panel). (D, E) The ratios of precipitated vinculin to total vinculin in B and C were quantified from three independent experiments using ImageJ. The asterisks indicate significant differences compared with WT vinculin using Bonferroni’s test (*P<0.05, **P<0.01).

Replacement of isoleucine 997 with alanine (IA) or valine 1001 with alanine (VA) in Vt reduces the affinity to actin [18, 22]. Thus, we first confirmed the binding of these mutants to filamentous actin (F-actin) using an *in vitro* actin co-sedimentation assay. Because purified wild-type (WT) vinculin is an inactive conformation, only a small amount of vinculin was coprecipitated with F-actin (Fig 1A). The addition of the α-helical vinculin-binding sites 3 of talin (VBS3(talin)), which induces the activation of vinculin [16], increased the amount of co-precipitated vinculin in a dose-dependent manner. Forty percent of vinculin was coprecipitated with F-actin when 25 μM VBS3(talin) was added (Fig 1B and 1D). T12 mutant vinculin, which is partially activated as a result of reduced Vh-Vt interaction [14], was efficiently coprecipitated. In contrast, neither the IA nor the VA mutation reduced the coprecipitation of vinculin with F-actin under these conditions. Another VBS derived from *Shigella* invasin IpaA (VBS(IpaA)) activates vinculin more efficiently than VBS3(talin) [31]. Thus, we also examined VBS(IpaA)-activated vinculin. VBS(IpaA) increased the coprecipitated WT vinculin to 84%. The IA and VA mutations reduced the coprecipitation of vinculin to 42% and 53%, respectively, as previously reported [18, 22]. Given that the vinculin tail domain has two actin-binding surfaces [18–20], these results suggest that the IA and VA mutations perturb one of the actin-binding surfaces, but the other binding surface may still bind actin. Although IA or VA mutation did not affect the binding of VBS3(talin)-activated vinculin to actin *in vitro*, these mutation are known to have effects on vinculin behaviors in cells [18, 22], indicating that vinculin is “fully” activated by a combination of talin with other binding partners in cells. In addition, the IA/VA double mutation did not further suppress the coprecipitation (Fig 1C and 1E). Thus, we used the IA and VA mutants as actin binding-deficient mutants for the analysis in cells in subsequent experiments.

### Subcellular localization and cell area

**Fig 2:**
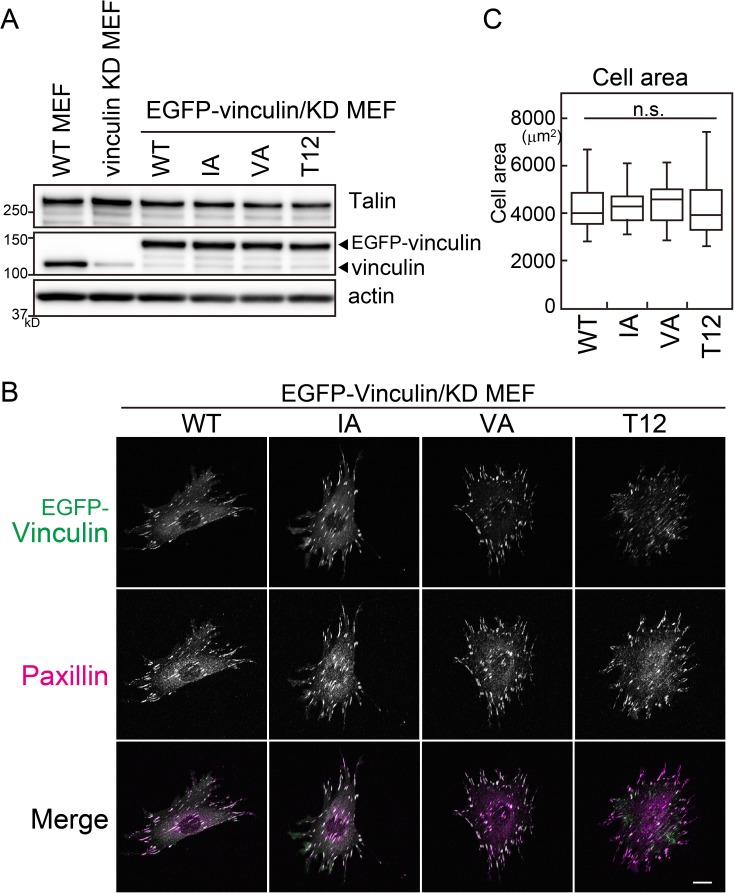
Expression levels and subcellular localization of vinculin mutants. (A) Expression levels of endogenous and GFP-tagged vinculin. Cell lysates were isolated from WT cells, vinculin KD cells, or vinculin KD cells expressing GFP-tagged vinculins. Talin and β-actin were used as loading controls. (B) Immunofluorescence analysis of cells expressing each mutant using the indicated antibodies. Scale bar: 20 μm. Fifty individual cells from two separate experiments were photographed for each condition. (C) Cell area. The values represent the means ± S.E.M. One-way ANOVA, Scheffe’s test for multiple comparisons (n = 50; n.s., non-significant).

EGFP-tagged WT or mutant vinculins were expressed in vinculin KD MEFs. As shown in Fig 2A, the expression levels of exogenous vinculin were comparable to that of endogenous vinculin. Thus, these cells are suitable for the analysis of the physiological function of vinculin. Under these conditions, WT and all mutant vinculin molecules were localized at focal adhesions and colocalized with another focal adhesion protein, paxillin (Fig 2B). The cell areas were also comparable, although a large dispersion was detected in the cell area of T12-expressing cells (Fig 2C). These results suggest that actin binding to the surface of vinculin containing I997V1001 (I/V surface) is dispensable for the localization to focal adhesions and for cell spreading.

### CSB resistance on substrates with physiological levels of stiffness

**Fig 3:**
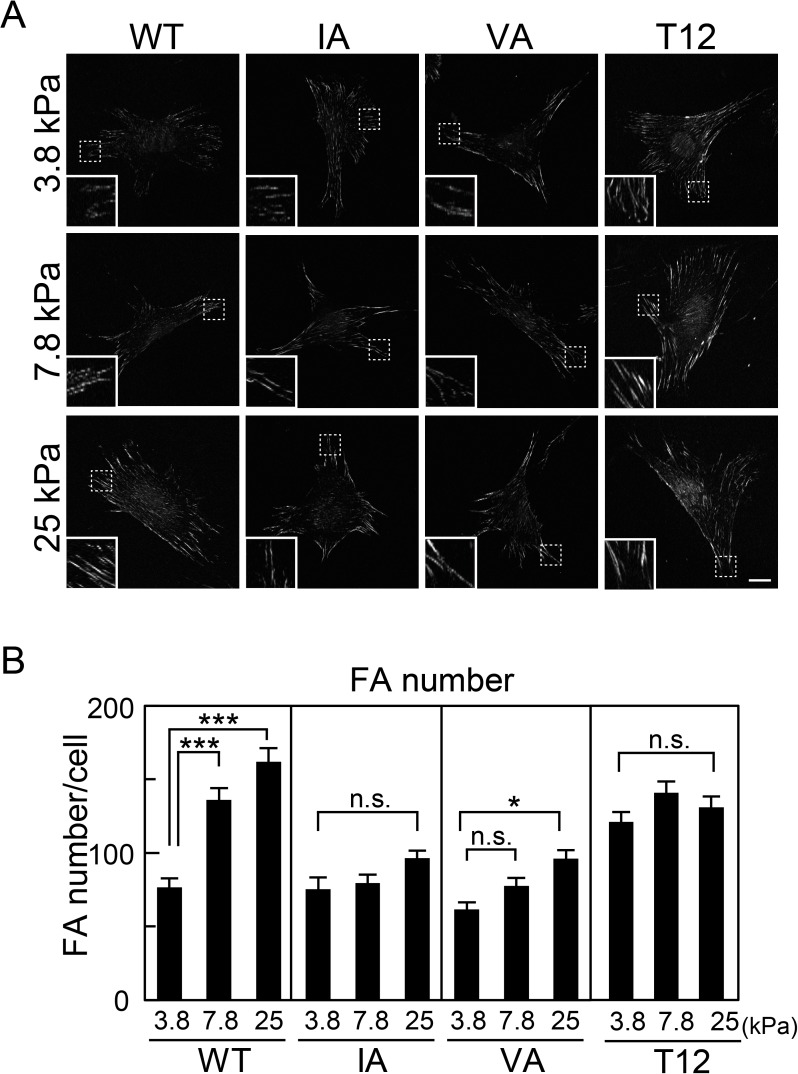
Visualization and quantification of CSB-resistant vinculin mutants. (A) GFP-vinculin-expressing cells cultured on polyacrylamide gels were treated with CSB, then fixed and visualized using GFP. The insets indicate higher-magnification images of the areas indicated with dashed boxes. Scale bar: 20 μm. Fifty individual cells from three separate experiments were photographed for each condition. (B) FA numbers were quantified from (A) using ImageJ. The values represent the means ± S.E.M. One-way ANOVA, Scheffe’s test (n = 50; *P<0.05, ***P<0.001 compared with 3.8 kPa gel; n.s., non-significant).

In a previous study, we showed that status of vinculin, its CSB resistance and its stable localization at FAs were regulated by ECM stiffness, and this regulation is thought to play a key role in sensing ECM stiffness [27]. CSB, which contains Triton X-100, removes cytoplasmic proteins, as well as proteins that are loosely attached to the cytoskeleton and to adhesion complexes from cells. To analyze the role of actin binding in CSB resistance, the cells were cultured on substrates with physiological levels of stiffness (3.8 ~ 25 kPa), corresponding to stiffness ranging from that of adipose tissue to that of cartilage and fibrotic tissue [32, 33]. Subsequently, immunostaining after CSB treatment was performed (Fig 3). The number of FAs containing CSB-resistant WT vinculin clearly increased in a stiffness-dependent manner as previously described [27]: 77 ± 6.2 and 162 ± 9.3 FAs containing CSB-resistant WT vinculin in each cell was detected on soft (3.8 kPa) and rigid (25 kPa) substrates, respectively. The total integrated density of CSB-resistant vinculin in each cell behaved similarly (S1 Fig). In contrast, the stiffness of the substrates only moderately affected the CSB resistance of IA and VA vinculin (Fig 3A and 3B). These differences in CSB resistance of vinculin are not due to the subcellular localization of vinculin or distribution of adhesions per se, since normal immunostaining without CSB treatment showed that the number and total integrated density of FAs containing WT or mutant vinculins was not increased on rigid substrates compared to on soft substrates under our experimental conditions (S2 Fig). These results indicate that actin binding to the I/V surface is necessary to regulate the CSB resistance of vinculin in a stiffness-dependent manner within a physiological range of substrate stiffness. Interestingly, on soft substrates, the number of FAs containing CSB-resistant T12 vinculin was significantly greater than that of FAs containing WT vinculin (Fig 3). Furthermore, stiffness did not affect the number or total integrated density of FAs containing CSB-resistant T12 vinculin, indicating that vinculin activation is involved in the stiffness-dependent regulation of vinculin resistance to CSB.

### CSB resistance on extremely rigid substrates

**Fig 4:**
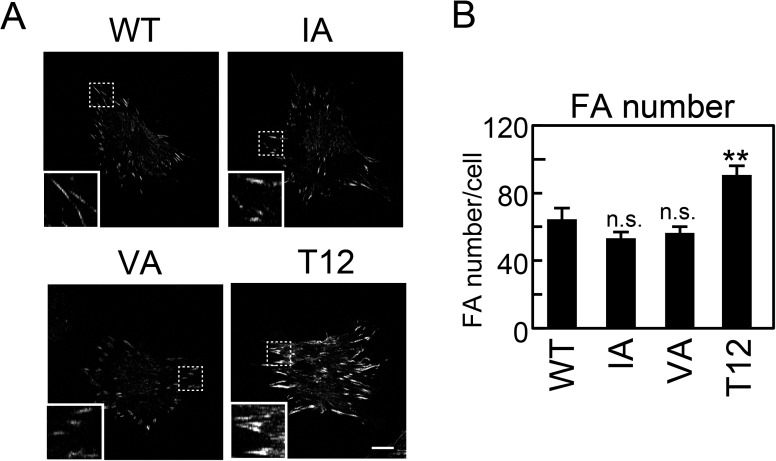
Visualization and quantification of CSB-resistant vinculin mutants. (A) GFP-vinculin-expressing cells cultured on glass coverslips were treated with CSB, then fixed and visualized using GFP. The insets indicate higher-magnification images of the areas indicated with dashed boxes. Scale bar: 20 μm. Thirty individual cells from two separate experiments were photographed for each condition. (B) FA numbers were quantified from (A) using ImageJ. The values represent the means ± S.E.M. Bonferroni’s test (n = 30; **P<0.01; n.s., non-significant).

Although there was clearly less CSB-resistant IA and VA vinculin than WT vinculin on rigid (25 kPa) substrates, there was slightly more than on soft (3.8 kPa) substrates (Fig 3). Thus, the cells were next cultured on glass coverslips (~GPa) to examine the effect of the IA and VA mutations on CSB resistance on extremely rigid substrates. As shown in Fig 4 and S3 Fig, CSB-resistant IA and VA vinculin was comparable to WT vinculin. Subcellular localization, as estimated based on normal immunostaining without CSB treatment, was also comparable (S2 Fig). Intriguingly, the number of FAs containing CSB-resistant T12 vinculin was significantly greater than that of FAs containing WT vinculin, indicating that on glass coverslips, not all WT vinculin is activated and CSB resistant. Together, these observations suggest that actin binding to the I/V surface is dispensable for vinculin CSB resistance on extremely rigid substrates.

### Stable localization of vinculin mutants on substrates with different levels of stiffness

The stable localization of vinculin at focal adhesions is regulated by ECM stiffness [27]. To investigate a role for actin binding on the stable localization of vinculin at FAs, FRAP analyses were performed using soft (3.8 kPa) or rigid (25 kPa) substrates. The immobile fraction of GFP-tagged WT vinculin, which represents the stably localized vinculin at FAs, was small (26 ± 2.8%) on soft substrates but large (37 ± 3.7%) on rigid substrates, as previously reported [27]. In contrast, the immobile fraction of IA vinculin was not affected by stiffness (28% and 31% on soft and rigid substrates, respectively). The immobile fraction of VA vinculin was also only slightly affected by stiffness. The T12 mutation increases the immobile fraction on glass substrates [34]. Interestingly, the immobile fraction of T12 vinculin was high, even on soft substrates, and the stiffness of the substrate did not affect this fraction (64% and 62% on soft and rigid substrates, respectively). These results suggest that actin binding to the I/V surface is required for regulating the stable localization of vinculin at FAs in a stiffness-dependent manner, and vinculin activation is involved in this regulation.

### T12/IA and T12/VA mutants on substrates with different levels of stiffness

To examine whether vinculin activation induced by T12 mutation is enough for the stable localization of and CSB resistance of vinculin at FAs, we introduced T12 mutation into the IA and VA background (T12/IA and T12/VA, respectively). FRAP analysis (S4 Fig) revealed that T12 mutation on the IA background increased the immobile fraction on either soft (62 ± 3.3%) or rigid (66 ± 2.3%) substrates: no significant difference between soft and rigid substrates were observed. T12/VA mutant showed similar results. These results suggest that vinculin activation is enough for stable localization of vinculin at FAs. As shown in S4 Fig, the number of FAs containing CSB-resistant vinculin were also increased by T12 mutation on the IA or VA background on either soft or rigid substrates, suggesting a critical role of vinculin activation in CSB resistance of vinculin. Interestingly, double mutations of T12 with IA or VA partially rescued the stiffness-dependent regulation of CSB resistance of vinculin. It is tempting to speculate that factors other than actin binding through I/V surface and vinculin activation have a role in stiffness-dependent regulation of CSB resistance of vinculin.

## Discussion

Vinculin has emerged as a major player in FA-mediated mechanotransduction. In a previous study, we showed that vinculin behavior, CSB resistance and stable localization at FAs depends on ECM stiffness [27]. In addition, vinculin and its binding partner, vinexin, are required for ECM stiffness-directed cell fate: differentiation to myoblasts and cell migration [24, 27]. Because intracellular tension plays a critical role in sensing ECM stiffness, vinculin binding to actin is important, but the role of actin binding in the ECM stiffness-dependent regulation of vinculin remains unclear. In the present study, we showed that actin binding-deficient mutations disrupted the ECM stiffness-dependent regulation of CSB resistance and the stable localization of vinculin. These results suggest that the vinculin-actin interaction is involved in FA-mediated mechanotransduction.

We used the IA and VA mutants, whose actin binding ability are reported to be reduced to half with vinculin stimulation with 100 μM IpaA. Decreased actin binding was also observed in the present study using 10 μM VBS(IpaA) as a vinculin activator (Fig 1). Interestingly, these mutations did not clearly reduce actin binding under mild activation conditions using VBS3(talin). Although the structure of Vt-actin is controversial [18–20], in all models, Vt binds to two actin protomers in F-actin through two surfaces. Interestingly, double substitutions at both surfaces that were based on one structural model showed a drastic reduction in actin binding [20]. Thus, the different effects of the IA and VA mutations on VBS(IpaA)- and VBS(talin)-activated vinculin can be explained using a model in which VBS(IpaA) and VBS3(talin) activate vinculin in different manners: VBS(IpaA) exposes two actin-binding surfaces, resulting in the efficient promotion of actin binding, whereas VBS3(talin) exposes only one surface that does not include the I/V residues.

Vinculin behavior, CSB resistance and stable localization at FAs, are regulated through ECM stiffness [27]. Here, we showed that the IA and VA mutations disrupted this regulation. Only a small fraction of these mutants showed CSB resistance and stable localization at FAs, even on rigid substrates, which is similar to observations on soft substrates (Fig 3). In contrast, a large fraction of activated mutant T12 showed CSB resistance and stable localization at FAs, even on soft substrates. These results suggest that actin binding to the I/V surface is important for ECM stiffness-dependent vinculin activation, resulting in stiffness-dependent vinculin regulation.

Intriguingly, the IA and VA mutations did not affect CSB resistance on extremely rigid (glass) substrates. The different effects of actin binding on the cell areas at physiological levels of stiffness and on extremely rigid substrates have also been reported [35]. One possibility to explain the discrepancy between the effects on the physiological levels of stiffness (gel substrates) and extremely rigid (glass) substrates is as follows: two tensile forces transmitted from two actin-binding surfaces are required for activation and CSB resistance of vinculin on the physiological level of stiffness because of the weak intracellular tension, while a high tensile force transmitted from one actin-binding surface on extremely rigid substrates is enough for them. Alternatively, features other than stiffness, such as surface chemical difference between glass and gel substrates, might modulate the effect of IA and VA mutation. When the actin-binding surface is determined in detail, it would be interesting to explore which actin-binding surfaces contribute to stiffness-dependent vinculin regulation at various levels of stiffness, from physiological to extremely rigid.

We showed that vinculin binding to actin is involved in the ECM stiffness-dependent regulation of vinculin behavior through vinculin activation. Simulation of molecular dynamics indicates that tension applied to vinculin changes its conformation, and further conformational changes then occur through binding with other molecules [36, 37]. In a previous study, we showed that vinexin α binding to vinculin is required for the ECM stiffness-dependent regulation of vinculin and cell migration [27]. Furthermore, vinexin α binding in the presence of F-actin *in vitro* induces vinculin activation [27]. Considering the combinatorial vinculin activation model [15], in which the simultaneous binding of more than two binding partners is required for vinculin activation, these observations suggest that both vinexin α- and actin-binding work co-operatively to induce vinculin activation in an ECM stiffness-dependent manner and contribute to the function of vinculin as a sensor for ECM stiffness. Under our experimental condition, WT as well as mutant vinculins localized at focal adhesions similarly even on soft (3.8 kPa) substrates (S2 Fig). It is observed that culturing cells on very soft substrates inhibits the FA formation [38, 39]. Thus, it is possible that actin binding also contributes to the FA formation or vinculin localization to FAs on very soft substrates that we have not tested. Future studies should examine this possibility.

**Fig 5:**
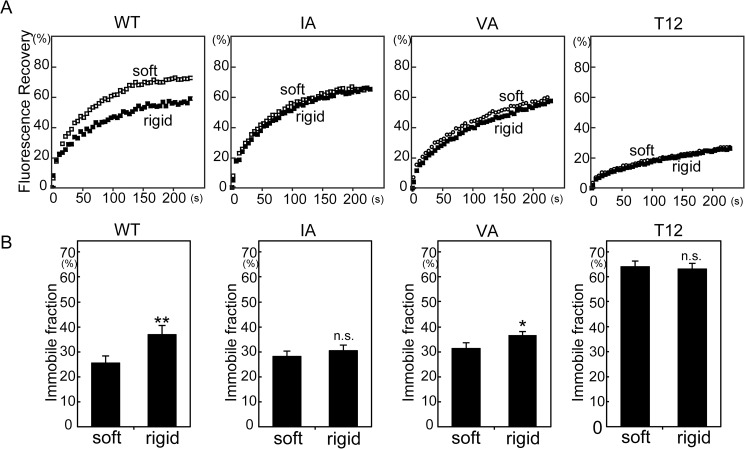
FRAP analysis of vinculin mutants on polyacrylamide gels. (A) GFP-vinculin-expressing cells were cultured on soft (3.8 kPa) or rigid (25 kPa) substrates. FRAP analysis was performed and normalized fluorescence recovery of EGFP-vinculin was plotted using data from two independent experiments (n = 80). (B) The immobile fractions were calculated from fitted curves. The values represent the means ± S.E.M. Bonferroni’s test (n > 30; *P<0.05, **P<0.02; n.s., non-significant).

Another interesting finding is the different effect of the T12 mutation on CSB resistance and immobile fraction of vinculin on rigid (25 kPa) gels (see Figs 3 and 5). CSB-resistant T12 vinculin was comparable to WT vinculin on rigid (25 kPa) gels, but the immobile fraction of T12 was much higher than that of WT vinculin. Both the CSB resistance and the immobile fraction of vinculin are regulated by binding to vinexin α and ECM stiffness [27]. Thus, these two parameters may represent the same status, such as the activated (open) conformation of vinculin. However, the different effect of the T12 mutation on CSB resistance and the immobile fraction of vinculin on rigid (25 kPa) gels indicates that this possibility is not likely. Rather, CSB resistance and the immobile fraction might reflect associations with specific proteins.

In summary, we used actin binding-deficient vinculin mutants and showed a critical role for the vinculin-actin interaction in the ECM stiffness-dependent regulation of vinculin behavior.

## Supporting information

S1 Fig Total integrated density of the CSB-resistant vinculin mutants, quantified from Fig 3A using ImageJ. The values represent the means ± S.E.M. One-way ANOVA, Scheffe’s test (n = 50; *P<0.05, **P<0.01, ***P<0.001; n.s., non-significant).
(PDF)

S2 Fig The number of FAs and the total integrated density of vinculin immunostaining without CSB treatment. GFP-vinculin-expressing cells cultured on polyacrylamide gels (A-C) or on coverslips (D-F) were fixed without CSB treatment and visualized using GFP. Fifty individual cells from three separate experiments (A) or thirty individual cells from two separate experiments (D) were photographed for each condition. (B, E) The number of FAs was quantified from A and D using ImageJ. (C, F) The total integrated density of GFP-vinculin quantified from A and D using ImageJ. The values represent the means ± S.E.M. One-way ANOVA, Scheffe’s test (B, C: n = 50; *P<0.05, **P<0.01 compared with 3.8 kPa gels, E, F: n = 30).
(PDF)

S3 Fig Total integrated density of CSB-resistant vinculin mutants quantified from Fig 4A using ImageJ. The values represent the means ± S.E.M. Bonferroni’s test (n = 30; **P<0.01; n.s., non-significant).
(PDF)

S4 Fig Analysis of T12/IA and T12/VA vinculin mutants. (A) FRAP analysis of T12/IA and T12/VA mutants on polyacrylamide gels. GFP-T12/IA- or GFP-T12/VA-expressing vinculin KD cells were cultured on soft (3.8 kPa) or rigid gel (25 kPa) substrates. FRAP analysis was performed and normalized fluorescence recovery of EGFP-vinculin was plotted using data from two independent experiments (n = 40). The immobile fractions were calculated from fitted curves. The values represent the means ± S.E.M. Bonferroni’s test (n = 40; *P<0.05, **P<0.02; n.s., non-significant). (B) Visualization and quantification of CSB-resistant T12/IA and T12/VA mutants. GFP-T12/IA or GFP-T12/VA-expressing cells cultured on polyacrylamide gels were treated with CSB, then fixed and visualized using GFP. Scale bar: 20 μm. Images were taken and analyzed as Fig 3. The values represent the means ± S.E.M. One-way ANOVA, Scheffe’s test (n = 50; *P<0.05, ***P<0.001 compared with 3.8 kPa gel; n.s., non-significant).
(PDF)
