# Supplementary figures and images for: Vinculin association with actin cytoskeleton is necessary for stiffness-dependent regulation of vinculin behavior
Source: PLoS One. 2017 Apr 7;12(4):e0175324. doi: 10.1371/journal.pone.0175324 (PMC5384775; doi:10.1371/journal.pone.0175324)

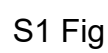

Supplement: S1 Fig — The values represent the means ± S.E.M. One-way ANOVA, Scheffe’s test (n = 50; *P<0.05, **P<0.01, ***P<0.001; n.s., non-significant). (PDF) [file pone.0175324.s001.pdf]

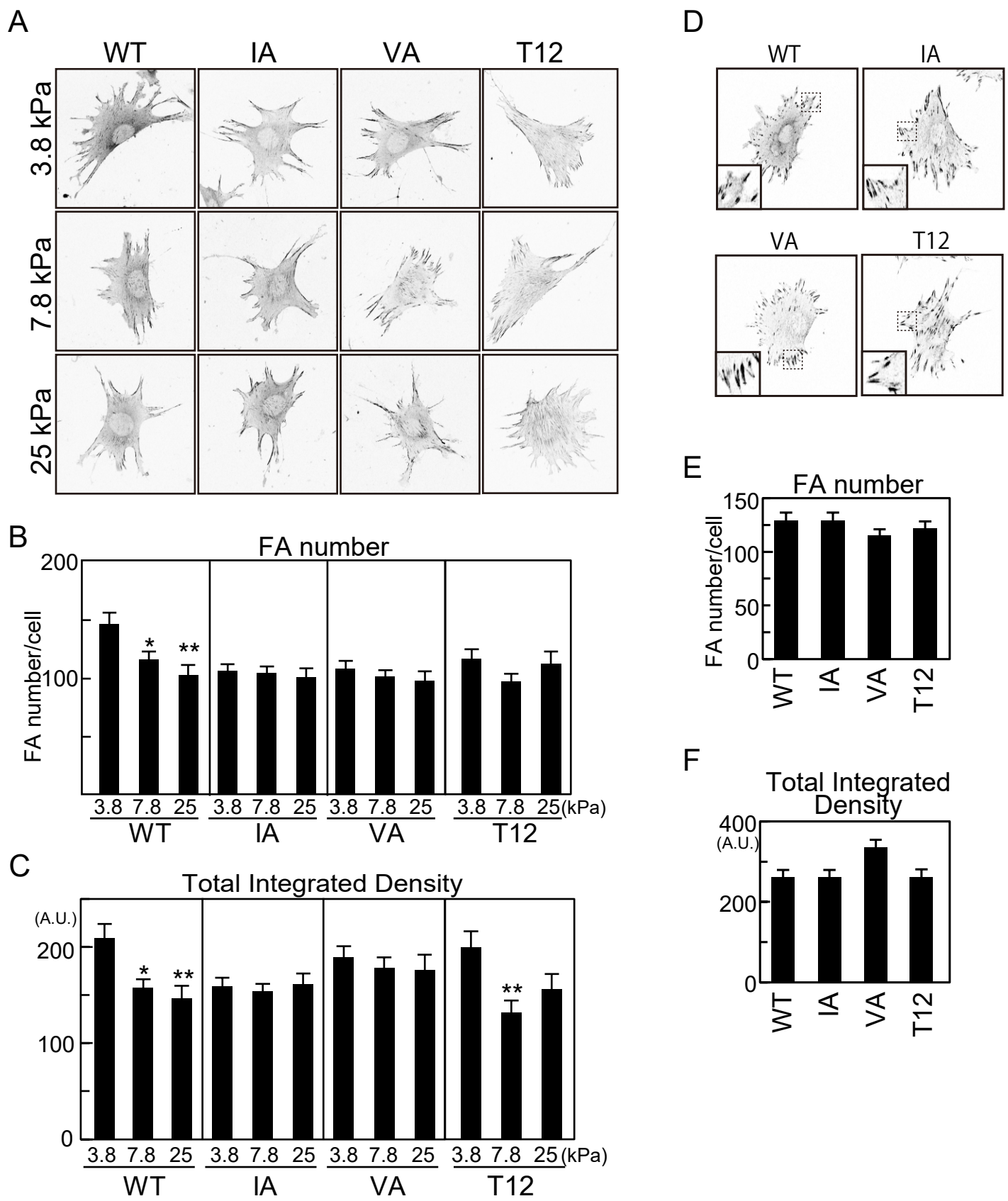

S2 Fig

Supplement: S2 Fig — GFP-vinculin-expressing cells cultured on polyacrylamide gels (A-C) or on coverslips (D-F) were fixed without CSB treatment and visualized using GFP. Fifty individual cells from three separate experiments (A) or thirty individual cells from two separate experiments (D) were photographed for each condition. (B, E) The number of FAs was quantified from A and D using ImageJ. (C, F) The total integrated density of GFP-vinculin quantified from A and D using ImageJ. The values represent the means ± S.E.M. One-way ANOVA, Scheffe’s test (B, C: n = 50; *P<0.05, **P<0.01 compared with 3.8 kPa gels, E, F: n = 30). (PDF) [file pone.0175324.s002.pdf]

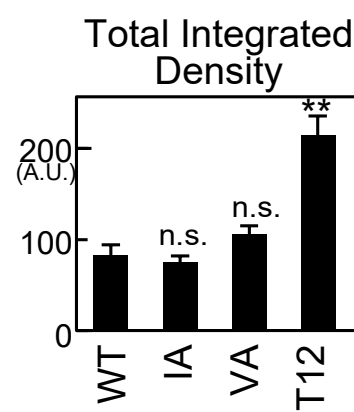

S3 Fig

Supplement: S3 Fig — The values represent the means ± S.E.M. Bonferroni’s test (n = 30; **P<0.01; n.s., non-significant). (PDF) [file pone.0175324.s003.pdf]

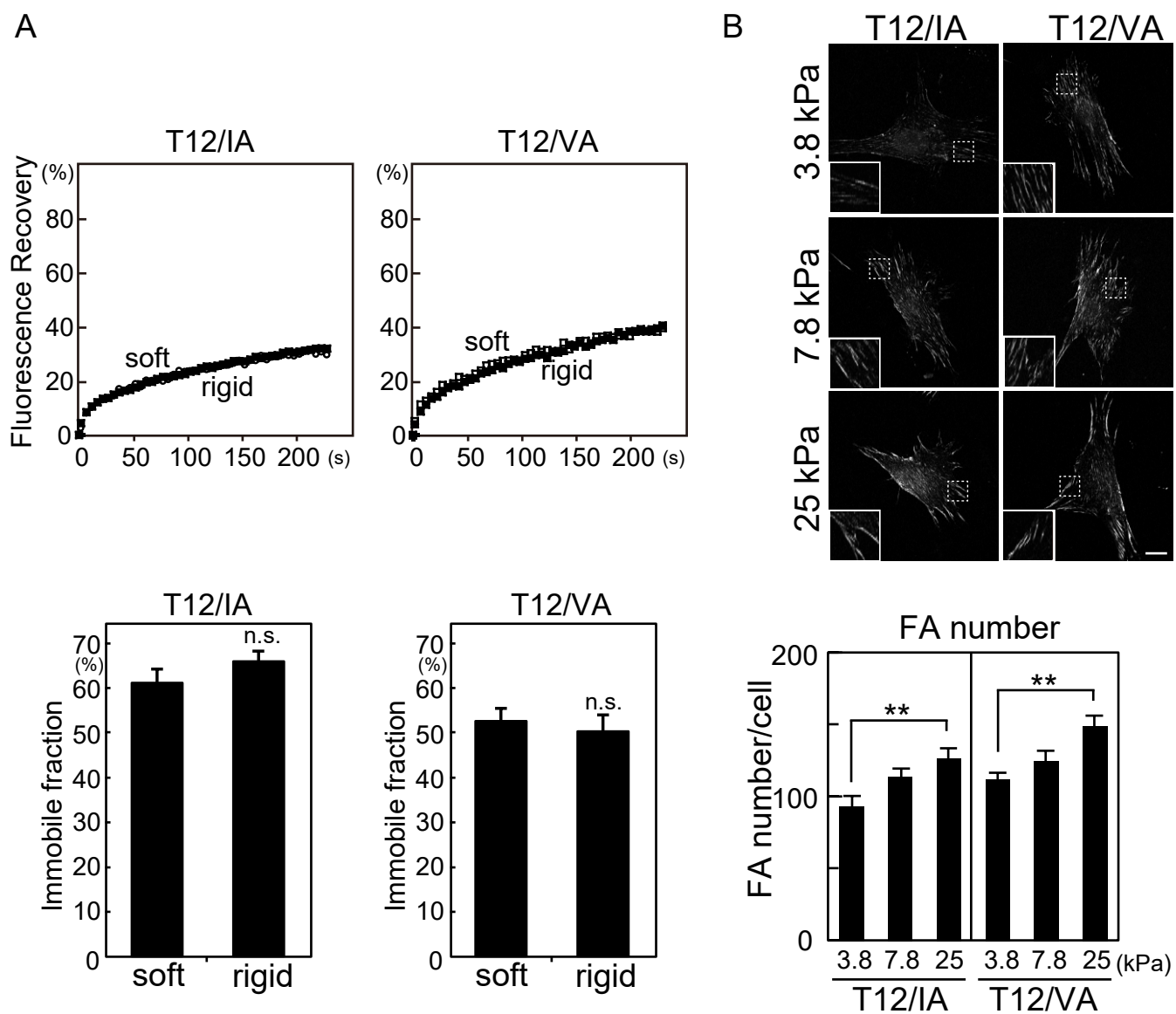

S4 Fig

Supplement: S4 Fig — (A) FRAP analysis of T12/IA and T12/VA mutants on polyacrylamide gels. GFP-T12/IA- or GFP-T12/VA-expressing vinculin KD cells were cultured on soft (3.8 kPa) or rigid gel (25 kPa) substrates. FRAP analysis was performed and normalized fluorescence recovery of EGFP-vinculin was plotted using data from two independent experiments (n = 40). The immobile fractions were calculated from fitted curves. The values represent the means ± S.E.M. Bonferroni’s test (n = 40; *P<0.05, **P<0.02; n.s., non-significant). (B) Visualization and quantification of CSB-resistant T12/IA and T12/VA mutants. GFP-T12/IA or GFP-T12/VA-expressing cells cultured on polyacrylamide gels were treated with CSB, then fixed and visualized using GFP. Scale bar: 20 μm. Images were taken and analyzed as Fig 3. The values represent the means ± S.E.M. One-way ANOVA, Scheffe’s test (n = 50; *P<0.05, ***P<0.001 compared with 3.8 kPa gel; n.s., non-significant). (PDF) [file pone.0175324.s004.pdf]
